# Comparing GPs’ antibiotic prescribing decisions to a clinical prediction rule: an online vignette study

**DOI:** 10.3399/BJGP.2020.0802

**Published:** 2023-02

**Authors:** Martine Nurek, Alastair D Hay, Olga Kostopoulou

**Affiliations:** Department of Surgery and Cancer, Imperial College London, London.; Centre for Academic Primary Care, Bristol Medical School: Population Health Sciences, University of Bristol, Bristol.; Department of Surgery and Cancer, Imperial College London, London.

**Keywords:** antimicrobial stewardship, decision support, general practice, primary care, STARWAVe

## Abstract

**Background:**

The ‘STARWAVe’ clinical prediction rule (CPR) uses seven factors to guide risk assessment and antibiotic prescribing in children with cough (Short illness duration, Temperature, Age, Recession, Wheeze, Asthma, Vomiting).

**Aim:**

To assess the influence of STARWAVe factors on GPs’ unaided risk assessments and prescribing decisions.

**Design and setting:**

Clinical vignettes administered to 188 UK GPs online.

**Method:**

GPs were randomly assigned to view 32 (out of a possible 64) vignettes online depicting children with cough. The vignettes comprised the seven STARWAVe factors, which were varied systematically. For each vignette, GPs assessed risk of deterioration in one of two ways (sliding-scale versus risk-category selection) and indicated whether they would prescribe antibiotics. Finally, GPs saw an additional vignette, suggesting that the parent was concerned. Mixed-effects regressions were used to measure the influence of STARWAVe factors, risk-elicitation method, and parental concern on GPs’ assessments and decisions.

**Results:**

Six STARWAVe risk factors correctly increased GPs’ risk assessments (*b*s_sliding-scale_≥0.66, odds ratios [ORs]_category-selection_≥1.75, *P*s≤0.001), whereas one incorrectly reduced them (short illness duration: *b*_sliding-scale_ −0.30, OR_category-selection_ 0.80, *P*≤0.039). Conversely, one STARWAVe factor increased prescribing odds (temperature: OR 5.22, *P*<0.001), whereas the rest either reduced them (short illness duration, age, and recession: ORs≤0.70, *P*s<0.001) or had no significant impact (wheeze, asthma, and vomiting: *P*s≥0.065). Parental concern increased risk assessments (*b*_sliding-scale_ 1.29, OR_category-selection_ 2.82, *P*≤0.003) but not prescribing odds (*P* = 0.378).

**Conclusion:**

GPs use some, but not all, STARWAVe factors when making unaided risk assessments and prescribing decisions. Such discrepancies must be considered when introducing CPRs to clinical practice.

## INTRODUCTION

Antimicrobial resistance (AMR) is a major threat to public health, with AMR infections claiming an estimated 700 000 lives per year globally.^1^ This figure is expected to increase to 10 million by 2050 if no action is taken.^1^ Prudent antibiotic prescribing is central to the UK’s 5-year national action plan to tackle AMR.^1^ Most NHS prescriptions are issued in primary care,^2^ often for childhood respiratory tract infections (RTIs).^3^^,^^4^ It is known that antibiotics offer limited benefits in such patients,^5^^–^7 but children are perceived as vulnerable and the risk of future deterioration leads to ‘defensive’ antibiotic use.^4^^,^^8^^–^10

Risk of future deterioration in children with RTIs can now be estimated with reasonable accuracy, using the validated STARWAVe clinical prediction rule (CPR):^4^

- **S**hort illness duration (≤3 days);
- **T**emperature (≥37.8 °C);
- **A**ge (<2 years);
- **R**ecession;
- **W**heeze;
- **A**sthma; and
- **V**omiting.

STARWAVe uses these seven factors to differentiate children at ‘very low’ (0.3%, ≤1 factor present), ‘normal’ (1.5%, 2–3 factors present), and ‘high’ (11.8%, ≥4 factors present) risk of admission to hospital within a month.^4^

Consistent with national guidelines,^11^ STARWAVe supports antimicrobial treatment only in high-risk patients. With only 3% of acute childhood RTIs falling into the high-risk category,^4^ widespread STARWAVe uptake could dramatically decrease unnecessary prescribing.

One way to increase uptake is to integrate STARWAVe into patients’ electronic health records. Indeed, within-consultation STARWAVe decision support has been incorporated into a complex behavioural intervention (currently at clinical trial) intended to improve management of childhood RTIs.^12^ It is known, however, that risk algorithms can be met with distrust, especially if they conflict with the practitioner’s own clinical judgement.^13^ It is therefore important to understand whether and how practitioners deviate from STARWAVe, and how they interpret its clinical factors.

## METHOD

### Research questions and hypotheses

The present study investigated whether and, if so, how GPs’ risk assessments and prescribing decisions differ from those suggested by STARWAVe. As this was an extension and improvement of a previous study^14^ (for more information see the authors’ approved protocol: https://osf.io/y8epw), the expectation was to replicate the previous findings, specifically:

- vomiting and wheeze would both be associated with higher risk assessments and antibiotic prescribing odds;
- younger patient age would be associated with higher risk assessments but would not influence prescribing; and
- shorter duration would be associated with lower risk assessments and prescribing odds.

**Table:** How this fits in

The STARWAVe (Short illness duration, Temperature, Age, Recession, Wheeze, Asthma, Vomiting) clinical prediction rule is a promising antimicrobial stewardship tool that could dramatically decrease unnecessary prescribing in children with respiratory tract infections. Present study findings suggest, however, that GPs do not spontaneously interpret patient characteristics, symptoms, and signs in accordance with STARWAVe, particularly in their prescribing decisions. Discordance between GPs and STARWAVe must be addressed if this tool is to bring about meaningful change in practice.

The authors had no hypotheses concerning the remaining three STARWAVe factors, that were not investigated in the previous study.

The current study also addressed three secondary research questions.

- Which of two risk-elicitation methods will maximise alignment between GPs’ risk assessments and prescribing decisions? The authors had no hypotheses in this regard.
- Which of two risk-elicitation methods will maximise alignment between GPs and STARWAVe? The authors had no hypotheses in this regard.
- How might parental concern influence risk assessments and prescribing decisions? The authors expected prescribing to increase when parental concern was present (versus absent) from a clinical vignette.^15^ The authors had no hypotheses as to whether/how parental concern might influence risk assessments.

### Design and materials

Materials were 64 vignettes depicting children with cough (see Supplementary Table S1). Each child was described in terms of the STARWAVe factors, which were manipulated in a half-fractional factorial design (Table 1). For face validity, patient sex was included. For external validity, vignettes were based on real patients (further details in protocol: https://osf.io/y8epw).

**Table 1.** Manipulation of STARWAVe factors in the present study

**Factor**	**Factor levels^a^**	**Factor range**
Age	1: <2 years	4 months to 6 years^b^
	0: ≥2 years	

Illness duration	1: ≤3 days	1 to 21 days^c^
	0: >3 days	

Temperature	1: parent reports severe fever in the last 24 h	N/A
	0: none	

Vomiting	1: parent reports moderate/severe vomiting in the last 24 h	N/A
	0: none	

Current asthma	1: present	N/A
	0: none	

Inter/subcostal recession	1: present on examination	N/A
	0: none	

Wheeze	1: present on examination	N/A
	0: none	

a *Factor levels were based on the STARWAVe clinical prediction rule, which assigns 1 point if the patient is: aged <2 years; if illness duration is ≤3 days; if the parent reports severe fever in the last 24 h; if the parent reports moderate or severe vomiting in the last 24 h; if the patient has current asthma; if inter/subcostal recession is present on examination; and if wheeze is present on examination.*

b *Most vignettes (83%) featured children who were aged 1–6 years, which was the interquartile range in the prognostic cohort study that gave rise to STARWAVe. The full range in the STARWAVe cohort study was 3 months to 16 years.*

c *Most vignettes (73%) had an illness duration of 3–10 days, which was the interquartile range in the STARWAVe cohort study. The full range in the STARWAVe cohort study was 0 28 days. N/A = not applicable.*

The vignettes were divided into two sets (set A and B) and each set was subdivided into two surveys. GPs were randomly assigned to either set A or B and saw the two surveys 24 h apart (order counterbalanced across GPs). The number of very-low, normal, and high-risk cases was consistent across sets and surveys (see Supplementary Table S2).

Seven ‘parental concern’ vignettes were also constructed, by adding the phrase ‘the parent is quite concerned’ to each of the seven very-low-risk cases (see Supplementary Table S3). Each GP saw one parental concern vignette, selected at random from the very-low-risk cases in their unseen set. This vignette was always presented last, so as not to influence responses to the ‘primary’ vignettes.

### Procedure

**Figure 1.**
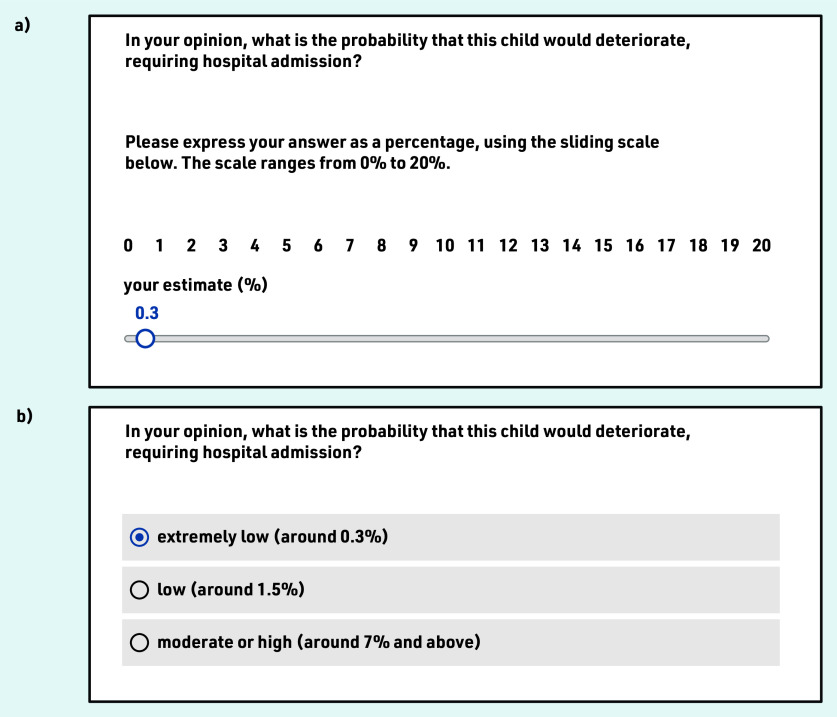
*Response scales used to elicit perceived risk of admission to hospital. Mock responses are provided for illustrative purposes: a) ‘0.3%’ on the sliding scale and b) ‘extremely low, around 0.3%’ on the category-selection scale.*

GPs received an invitation email, with a link to an expression of interest form (see Supplementary Appendix S1). Those who expressed interest were emailed a link to the study website. After providing consent and reading an introduction to the study (see Supplementary Appendix S2), GPs saw 16 vignettes in a random order. Per vignette, they were asked three questions:

1. ‘In your opinion, what is the probability that this child would deteriorate, requiring hospital admission?’ Half of the sample (randomly selected) expressed their answer as a percentage, using a sliding scale (Figure 1a). The scale was capped at 20% because the probability rarely exceeds 17% in this cohort.^4^ The rest selected between three risk categories (‘extremely low, around 0.3%’, ‘low, around 1.5%’, and ‘moderate or high, around 7% and above’; Figure 1b). These categories correspond to STARWAVe’s three levels of risk, but the STARWAVe labels (‘very low’, ‘normal’, ‘high’) were replaced by GP-appointed labels, elicited in a pilot study (see Supplementary Appendix S3). For consistency, the authors have adopted the terminology of level 1 (lowest), level 2 (middle), and level 3 (highest) risk, whether referring to GPs’ responses or STARWAVe categories.
2. ‘In your clinical judgment, what would be the best course of action?’ Three options were provided for patient management: ‘prescribe antibiotics’, ‘arrange to GP review within 24 h’, and ‘admit for paediatric assessment’. GPs could select all that applied (or none). When ‘prescribe antibiotics’ was selected, GPs were asked whether the antibiotic would be ‘immediate’ or ‘delayed’.
3. ‘Please enter any additional comments (optional)’.

GPs were emailed a link to a second survey 24 h later, comprising 16 previously unseen vignettes and one parental concern vignette. Response scales were identical to those seen in the first survey. The procedure is presented graphically in Supplementary Figure S1.

Surveys were hosted online using Qualtrics. After completing both surveys, GPs received £50 via bank transfer and the National Institute for Health Research Clinical Research Network (NIHR CRN) gave £50 to each GP’s practice. Data were collected April to October 2021. Departures from the approved protocol are reported in Supplementary Appendix S4.

### Analysis

#### Primary research question

To investigate the effect of the STARWAVe factors on risk assessments and prescribing decisions, three mixed-effects regression models were built, with random intercept and slope by participant:

- continuous risk assessments (cast on a sliding scale) were regressed on patient age (continuous), duration (continuous), vomiting, asthma, recession, temperature, and wheeze (0 = absent and 1 = present; linear model);
- risk-category selections (0 = level 1, 1 = level 2, and 2 = level 3) were regressed on the same (ordinal logistic model); and
- prescribing decisions were dichotomised (0 = no/delayed prescription and 1 = immediate prescription) in line with national guidelines (which treat ‘no prescription’ and ‘delayed prescription’ interchangeably)^11^ and regressed on the same factors (binary logistic model). For exploratory purposes, the model was repeated with a three-category ordinal dependent variable (0 = no prescription, 1 = delayed prescription, and 2 = immediate prescription; ordinal logistic model).

All models were repeated with age and duration treated as binary, in keeping with the STARWAVe CPR (age = 1 if <2 years, duration = 1 if ≤3 days, otherwise 0).

#### Secondary research questions

To measure the alignment between GPs’ risk assessments and prescribing decisions — and to compare this across risk-response modes — immediate prescriptions were classified as either ‘consistent’ or ‘inconsistent’ with the GP’s risk assessment. As prescribing was not the only management option available to GPs, each case was also classified as ‘appropriately’ or ‘inappropriately’ managed, relative to the GP’s risk assessment. (For full details of these classification systems see protocol: https://osf.io/y8epw). These two variables were then regressed on risk-response mode, using mixed-effects, random-intercept binary logistic regression.

To measure GPs’ alignment with STARWAVe — and compare this across risk-response modes — risk assessments, immediate prescriptions, and broader management decisions were each classified as either ‘consistent’ or ‘inconsistent’ with STARWAVe (full details in protocol: https://osf.io/y8epw), and regressed on response mode using mixed-effects, random-intercept binary logistic regression.

To assess the impact of parental concern, only STARWAVe-identified level 1 cases (with and without parental concern) were selected and three mixed-effects, random-intercept regression models were built. First, risk assessments were regressed on parental concern (0 = absent and 1 = present), separately for those who used a sliding scale (linear model) versus category selection (ordinal logistic model). Thereafter, all prescribing decisions (0 = no/delayed prescription and 1 = immediate prescription) were regressed on parental concern (binary logistic model). Parental concern vignettes were not included in any other analyses.

Analyses were performed using Stata/MP (version 17.0). Mixed-effects regressions were conducted using the ‘mixed’ (linear), ‘melogit’ (binary), and ‘meologit’ (ordinal) commands.^16^ Violations of the proportional odds assumption were addressed using the ‘gologit2’ command.^17^^,^^18^

### Participants

GPs practising in the UK were eligible to participate, as were ST3/4 GP registrars. Participants were recruited via the NIHR CRN, who circulated the invitation email to practices across England.

Power analyses were conducted for each hypothesis (see Supplementary Appendix S5). These suggested that 429 GPs were needed to assess the effect of the STARWAVe factors on categorical risk assessments and 88 GPs for the continuous risk assessments. As 429 GPs was not feasible (GPs being a difficult-to-reach population), the authors deemed it practical to recruit two groups of 88 GPs (one per risk-response scale; *n* = 176 in total). Assuming a dropout rate of 2%, the aim was to recruit an additional four, yielding *n* = 90 per risk-response scale.

## RESULTS

In total, 306 GPs completed the expression of interest form. This is higher than the intended sample size because, in the authors’ experience, those who express interest do not always go on to complete the study. Indeed, only 199 completed the first survey (65%); of these 188 went on to complete the second survey (94%). The 11 GPs who did not complete the second survey were excluded from the analysis. Participant characteristics appear in Table 2.

**Table 2.** Participant characteristics per risk-response mode and overall

**Characteristic**	**Risk assessed on a sliding scale (*n* = 94)**	**Risk assessed via category selection (*n* = 94)**	**Total (*N* = 188)**
**Grade, *n* (%)**			
Qualified GP	86 (91)	81 (86)	167 (89)
GP registrar	8 (9)	13 (14)	21 (11)

**Number of years since GP** **qualification, mean (SD), range**	10.2 (8.9), 0–35	11.3 (9.4), 0–38	10.7 (9.1), 0–38

**Level of training (trainees), *n* (%)**			
ST3	6 (6)	11 (12)	17 (9)
ST4	2 (2)	2 (2)	4 (2)

**Diploma in Child Health,** ***n* (%)**			
Yes	15 (16)	18 (19)	33 (18)
No	79 (84)	76 (81)	155 (82)

**Member/Fellow of Royal College of Paediatrics and Child Health,** ***n* (%)**			
Yes	1 (1)	2 (2)	3 (2)
No	93 (99)	92 (98)	185 (98)

**Self-reported confidence when** **assessing sick children,^a^ mean (SD), range**	1.7 (0.6), 0–3	1.9 (0.5), 0–3	1.8 (0.6), 0–3

**Vignettes seen,** ***n* (%)**			
Set A, survey 1 first	24 (26)	24 (26)	48 (26)
Set A, survey 2 first	23 (24)	23 (24)	46 (24)
Set B, survey 1 first	23 (24)	24 (26)	47 (25)
Set B, survey 2 first	24 (26)	23 (24)	47 (25)

a *Self-reported confidence when assessing sick children was measured on a four-point scale (0 = ‘I seldom feel confident’, 1 = ‘I feel confident sometimes’, 2 = ‘I feel confident most of the time’, and 3 = ‘I always feel confident’). SD = standard deviation.*

### Descriptive statistics

**Figure 2.**
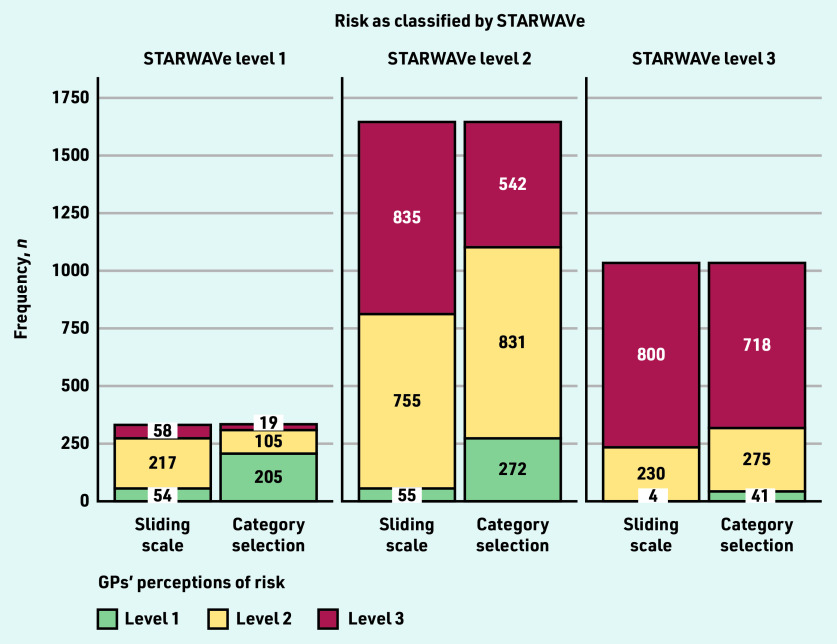
*GPs’ risk assessments (level 1 versus level 2 versus level 3) by STARWAVe risk classification and risk-response mode. Risk assessments cast on a 0%–20% sliding scale were classified as level 1 if <1%, level 2 if 1%–6.9%, or level 3 if ≥7%. Risk assessments cast via category selection were classified as level 1 if ‘extremely low, around 0.3%’ was selected, level 2 if ‘low, around 1.5%’ was selected, or level 3 if ‘moderate or high, around 7% and above’ was selected. The number of cases that STARWAVe classified as level 1, level 2, and level 3 was 329, 1645, and 1034, respectively.*

On average — excluding parental concern vignettes — the sliding-scale group estimated risk of admission to hospital to be 4.1% (standard deviation [SD] 3.92), 7.9% (SD 5.32), and 11.9% (SD 5.50) for cases classified by STARWAVe as level 1, level 2, and level 3, respectively (data not shown). Figure 2 compares sliding-scale group risk assessments (categorised as level 1 if <1%, level 2 if 1%–6.9%, or level 3 if ≥7%) with those of the category-selection group and STARWAVe. Data suggests that the sliding-scale group tended to overestimate risk, frequently assigning a risk level of 2 to cases that STARWAVe deemed level 1 (66%, *n* = 217/329), and a risk level of 3 to cases that STARWAVe deemed level 2 (51%, *n* = 835/1645). The category-selection group appeared less likely to overestimate risk, with most GPs selecting level 1 for STARWAVe-identified level 1 cases (62%, *n* = 205/329) and level 2 for STARWAVe-identified level 2 cases (51%, *n* = 831/1645).

**Figure 3.**
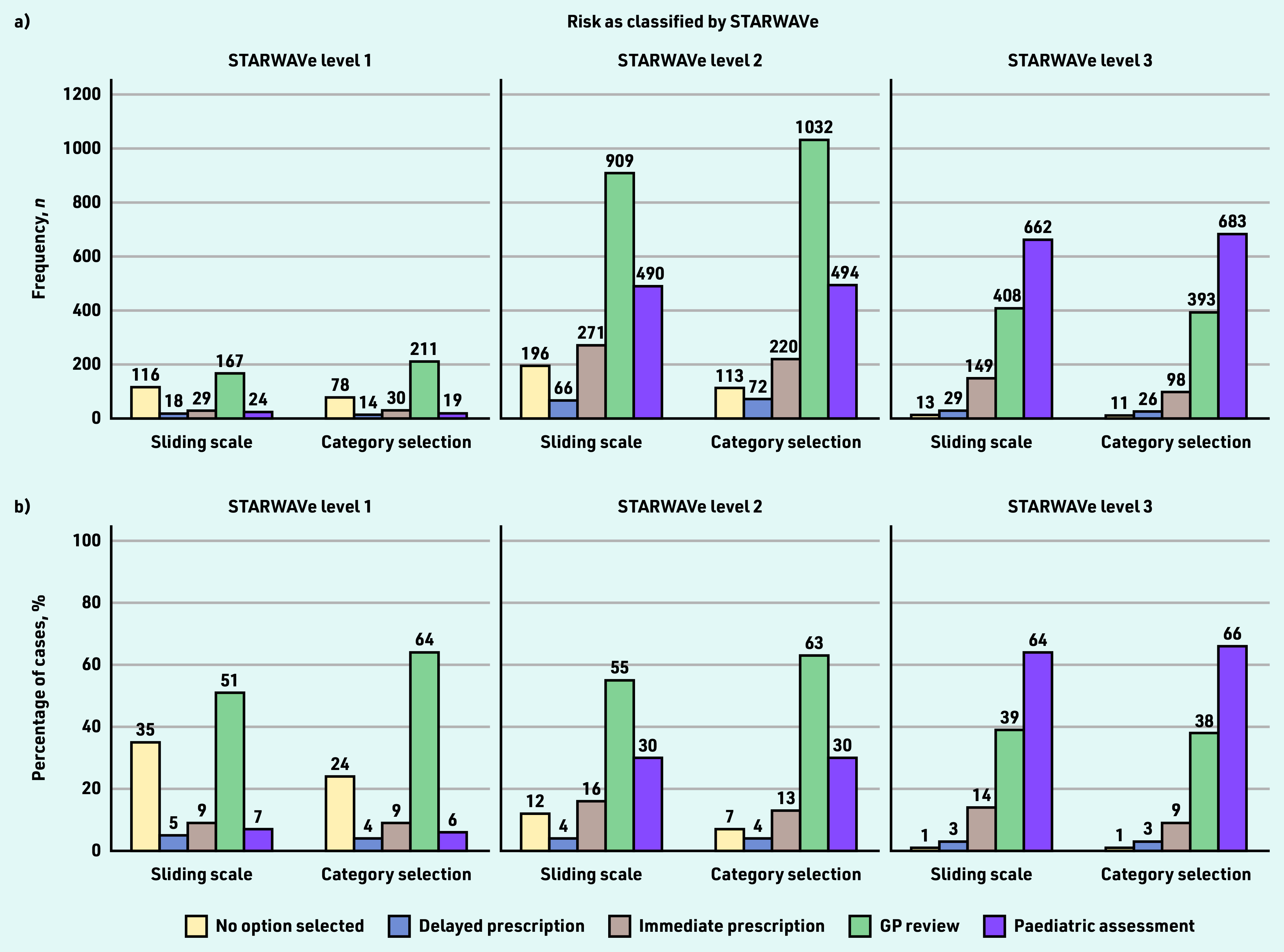
*GPs’ selections for patient management, by STARWAVe risk classification and risk-response mode. The Figure displays a) the number and b) proportion of times that each option for patient management was chosen. Participants could select multiple options (or none) therefore percentages do not sum to 100. The total number of cases classified by STARWAVe as level 1, level 2, and level 3 was 329, 1645, and 1034, respectively. The appropriate management strategies (according to STARWAVe) are: no action in level 1 cases; no action or delayed prescription in level 2 cases; 24 hr GP review and/or immediate prescription in level 3 cases. Admission for paediatric assessment is not recommended by STARWAVe but is a reasonable strategy in more severe cases.*

GPs’ management selections are shown in Figure 3. In STARWAVe-identified level 1 cases, the commonest course of action was to review the patient (selected 51% [*n* = 167/329] and 64% [*n* = 211/329] of the time in the sliding-scale and category-selection groups, respectively), although many preferred to take no action at all (35% [*n* = 116/329] and 24% [*n* = 78/329], respectively).

In STARWAVe-identified level 2 cases, the commonest action was again GP review (55% [*n* = 909/1645] and 63% [*n* = 1032/1645] in the sliding-scale and category-selection groups, respectively), although a substantial minority opted to admit the patient for paediatric assessment (30% [*n* = 490/1645 and *n* = 494/1645] in both the sliding-scale and category-selection groups) (Figure 3).

Admission for paediatric assessment was the most-selected option for STARWAVe-identified level 3 cases (64% [*n* = 662/1034] and 66% [*n* = 683/1034] in the sliding-scale and category-selection groups, respectively), although GP review remained prominent (39% [*n* = 408/1034] and 38% [*n* = 393/1034], respectively) (Figure 3).

Across the board, antibiotics were infrequently prescribed and tended to be immediate (15% and 12% of all cases in the sliding-scale and category-selection groups, respectively) rather than delayed (4% in both groups).

### Primary research question

The effect of STARWAVe risk factors on GPs’ risk assessments is shown in Table 3 (columns 1 and 2). In keeping with STARWAVe (green cells), presence of fever (temperature), recession, wheeze, asthma, and vomiting significantly increased risk assessments, as did younger age (that is, older age reduced them). Inconsistent with STARWAVe (red cells), longer duration significantly increased risk assessments.

**Table 3.** Effect of STARWAVe risk factors on GPs’ risk assessments and prescribing decisions^a^

**STARWAVe factor**	**Risk assessments**		**Antibiotic prescriptions, OR (95% CI)**
	**Sliding scale, *b* (95% CI)**	**Category selection, OR (95% CI)**	
Duration (ascending)	0.09^b^ (0.04 to 0.13)	1.04^c^ (1.01 to 1.06)	1.19^b^ (1.15 to 1.23)
Temperature	2.53^b^ (2.16 to 2.90)	5.28^b^ (4.09 to 6.81)	7.18^b^ (5.33 to 9.68)
Age (ascending)	−0.26^b^ (−0.35 to −0.17)	0.88^b^ (0.83 to 0.93)	1.17^b^ (1.10 to 1.24)
Recession	5.29^b^ (4.77 to 5.81)	55.55^b^ (39.28 to 78.54)	0.45^b^ (0.33 to 0.62)
Wheeze	2.45^b^ (2.15 to 2.75)	6.53^b^ (5.11 to 8.35)	0.85 (0.68 to 1.07)
Asthma	0.64^b^ (0.40 to 0.88)	2.07^b^ (1.73 to 2.47)	1.06 (0.85 to 1.33)
Vomiting	1.66^b^ (1.37 to 1.95)	3.29^b^ (2.65 to 4.08)	0.82 (0.66 to 1.03)

a *Data in green is consistent with STARWAVe, data in red is inconsistent with STARWAVe, and data in orange is of no significant impact. Age and duration were treated as continuous in these models; when treated as binary, findings did not change (see Supplementary Table S4). Column 1 (risk assessments cast on a sliding scale): the model included random slopes for all seven STARWAVe factors. Column 2 (risk assessments cast via category selection): a model that included random slopes for all seven STARWAVe factors would not converge, therefore the authors identified the random slopes that best improved model fit and added them to the model progressively until non-convergence occurred (see Supplementary Appendix S6). The final model contained random slopes for recession, temperature, wheeze, and vomiting. Three factors violated the proportional odds assumption (duration, wheeze, and vomiting): their effect reduced as risk assessments increased (see Supplementary Appendix S7). Column 3 (prescriptions): the model would not converge with random slopes for all STARWAVe factors, therefore the authors identified and included the random slopes that best improved model fit (see Supplementary Appendix S8). The final model contained random slopes for all STARWAVe factors except age. When prescriptions were treated as a three-category ordinal variable (0 = no prescription, 1 = delayed prescription, and 2 = immediate prescription), findings did not change (see Supplementary Table S5).*

b P≤*0.001.*

c P≤*0.05.*

*OR = odds ratio.*

Table 3 also shows the effect of the STARWAVe factors on prescribing decisions. Consistent with STARWAVe (green cell), temperature significantly increased prescribing odds; inconsistent with STARWAVe (red cells), longer duration and older age increased them, whereas recession reduced them. Wheeze, asthma, and vomiting had no significant impact (orange cells) on prescribing odds.

### Secondary research questions

#### Alignment between GPs’ risk assessments and prescribing decisions

Half of GPs’ prescriptions were inconsistent with their own risk assessments; that is, administered in cases that they perceived to be level 1 or level 2 (51%, *n* = 407/797). This occurred significantly more often in the category-selection group (64% of prescriptions, *n* = 223/348) than the sliding-scale group (41%, *n* = 184/449; odds ratio [OR] 4.25, 95% confidence interval [CI] = 2.16 to 8.39, *P*<0.001). More broadly, management selections were inconsistent with subjective risk assessments 42% of the time (*n* = 2549/6016), again more often in the category-selection group (50%, *n* = 1513/3008) than the sliding-scale group (34%, *n* = 1036/3008; OR 2.11, 95% CI = 1.65 to 2.69, *P*<0.001; see Supplementary Figure S2).

#### Alignment between GPs and STARWAVe

Risk assessment was inconsistent with STARWAVe 44% of the time (*n* = 2653/6016), although this was less frequent in the category-selection group (42% inconsistent, *n* = 1254/3008) than the sliding-scale group (47% inconsistent, *n* = 1399/3008; OR 0.82, 95% CI = 0.73 to 0.92, *P* = 0.001) (Figure 2). As Figure 2 suggests, the sliding-scale group tended to overestimate risk (37% of cases, *n* = 1110/3008) rather than underestimate it (10%, *n* = 289/3008), whereas the category-selection group did both (22% and 20%, *n* = 666/3008 and *n* = 588/3008).

Although risk assessment was better aligned with STARWAVe in the category-selection group, prescribing decisions were not. Most immediate prescriptions (69%, *n* = 550/797) were unnecessary according to STARWAVe, and this proportion did not differ by risk-response mode (72%, *n* = 250/348 versus 67%, *n* = 300/449; OR 1.28, 95% CI = 0.92 to1.77, *P* = 0.145). In fact, the category-selection group was more likely than their counterparts to manage the patient inappropriately, according to STARWAVe (59%, *n* = 1774/3008 versus 55%, *n* = 1656/3008; OR 1.17, 95% CI = 1.05 to 1.32, *P* = 0.006) (Figure 3).

#### Parental concern

Adding parental concern to STARWAVe-identified level 1 vignettes significantly increased risk assessments in both groups (*b*_sliding-scale_ 1.29, 95% CI = 0.44 to 2.14, *P* = 0.003; OR_category-selection_ 2.82, 95% CI = 1.77 to 4.49, *P*≤0.001; proportional odds assumption met with χ^2^(1) = 0.01, *P* = 0.938). In the sliding-scale group, the mean risk assessments were 5.3% (concern present) versus 4.1% (absent); in the category-selection group, the median/modal risk assessments were level 2 (concern present) versus level 1 (absent) (data not shown).

Parental concern did not influence prescribing (OR 0.74, 95% CI = 0.39 to 1.44, *P* = 0.378) but did increase the odds of GP review (OR 2.88, 95% CI = 1.90 to 4.36, *P*<0.001) and paediatric assessment (OR 2.82, 95% CI = 1.72 to 4.63, *P*<0.001). When parental concern was present in these vignettes, GPs prescribed 7% of the time, reviewed the patient 76% of the time, and admitted the patient 17% of the time (see Supplementary Table S6) whereas when parental concern was absent in these vignettes, GPs prescribed 9% of the time, reviewed the patient 57% of the time, and admitted the patient 7% of the time.

## DISCUSSION

### Summary

The present study measured the effect of STARWAVe risk factors, risk elicitation method (sliding scale versus category selection), and parental concern on GPs’ risk assessments and antibiotic prescribing decisions in children presenting with cough and RTI. Six STARWAVe risk factors correctly increased GPs’ risk assessments (temperature, age, recession, wheeze, asthma, and vomiting), whereas one incorrectly reduced them (short illness duration). Conversely, only one STARWAVe risk factor increased prescribing odds (temperature); the rest either reduced them (short illness duration, age, and recession) or had no significant impact (wheeze, asthma, and vomiting). Risk-elicitation method (sliding scale versus category selection) and parental concern (present versus absent) increased GPs’ risk assessments, but not their prescribing odds.

### Strengths and limitations

Methodological rigor was the guiding principle of this study, which improved on a previous one^14^ by:

- basing patient vignettes on real patients;
- varying all seven STARWAVe factors systematically;
- testing the effects of two new risk-elicitation methods;
- ensuring a comprehensive and clinically plausible set of options for patient management; and
- testing the effect of a non-clinical factor (parental concern).

More broadly, the present study used a robust experimental approach that is under-represented in this area of research and predefined the hypotheses, procedure, and analyses in an approved protocol.

Despite the authors’ attempts to improve ecological validity, patients appeared ‘on paper’ rather than in person. The authors therefore appreciate that some of the findings may fail to translate to clinical practice, where situational variables such as time pressure are known to play a role.^8^^,^^9^^,^^19^^–^22 Presently, for example, GPs elected to review (within 24 h) roughly half the patients seen; whether they would or could review these patients when faced with the pressures of routine practice is questionable.

The authors are also aware that analyses limited to the category-selection group (Table 3, column 2) were underpowered and therefore exploratory. Although findings were comparable with those of the sliding-scale group, they would benefit from replication in a larger study. Future work might also assess whether present findings extend to other primary care clinicians who manage childhood RTIs, including nurses, paramedics, and pharmacists.

### Comparison with existing literature

Presently, GPs’ risk assessments were aligned with STARWAVe roughly half of the time, though degree of alignment varied by risk response mode (sliding scale 53% versus category selection 58%). Interestingly, risk-response mode also influenced GPs’ deviations from STARWAVe: the sliding-scale group tended to overestimate (versus underestimate) risk, while the category-selection group did both. In a previous study,^14^ the authors employed a third risk-response mode and observed yet a third deviation from STARWAVe (systematic underestimation of risk). Taken together, these findings suggest that risk-elicitation method can influence risk perceptions (‘reactivity’^23^^,^^24^).

The prescribing rate was low in the present study (13%), with GPs preferring further assessment (review/referral) to immediate prescribing. Still, many prescriptions were unnecessary relative to GPs’ own risk assessments (51%) and STARWAVe’s (69%). These findings were consistent across risk-response modes and consistent with the authors’ previous study,^14^ where prescriptions (administered 15% of the time) were usually unnecessary relative to GPs’ (78%) and STARWAVe’s (83%) assessment of risk.

These findings suggest a disconnect between risk assessments and prescribing decisions, which was also apparent in GPs’ interpretation of STARWAVe’s risk factors. Younger patient age and recession increased GPs’ risk assessments (in keeping with STARWAVe) but reduced prescribing odds. Vomiting, asthma, and wheeze likewise increased risk assessments (in keeping with STARWAVe) but did not affect prescribing. Only long illness duration and temperature increased both risk perceptions and prescribing, but the former runs counter to STARWAVe (which posits short illness duration as a risk factor).

Notably, the effect of the STARWAVe factors on GPs’ risk assessments was consistent with 1) the authors’ previous study,^14^ and 2) STARWAVe itself (with only one factor — illness duration — incorrectly informing GPs’ risk assessments). In contrast, the effect of the STARWAVe factors on GPs’ prescribing decisions was not consistent with the authors’ previous study^14^ or STARWAVe (with only one factor — temperature — correctly informing GPs’ decisions). GPs’ interpretation of the STARWAVe factors would thus appear to be stable and largely appropriate when it comes to risk assessment, but variable and largely inappropriate when it comes to prescribing decisions.

In very-low-risk cases (level 1), parental concern raised GPs’ risk assessments but not prescribing odds, speaking again to the disconnect between risk assessment and prescribing. The non-association between parental concern and prescribing was somewhat surprising, given that parental pressure for antibiotics is known to increase their likelihood.^9^^,^^15^^,^^19^^,^^25^^,^^26^ Parental concern, however, is not specific to antibiotics, and it did propel GPs to take other types of action including 24 h review and admission for paediatric assessment (both unnecessary in very-low-risk cases).

### Implications for research and practice

If STARWAVe is to be provided as a decision aid to GPs, then several issues need to be addressed. First, risk of admission to hospital guides prescribing in the STARWAVe model; the current findings suggest that this may not be true for GPs. Consequently, a STARWAVe-based decision aid may fail to bring about meaningful change. As mentioned in the Introduction, a multicomponent intervention that includes STARWAVe decision support is currently at clinical trial,^12^ but its success/failure cannot confidently be attributed to a single component. Therefore, further work is needed to isolate the effect of STARWAVe on prescribing practices.

Second, decision aid developers will need to consider how best to present risk estimates to GPs. The present findings suggest that the risk-elicitation format can influence explicit risk assessments. Risk assessments cast on a sliding scale were inflated relative to those cast via category selection, but they were also better aligned with GPs’ own management decisions (suggesting a certain ‘fidelity’ or ‘construct validity’) and STARWAVe’s recommended management pathways (suggesting potential normative value). Presenting risk scores as point-estimates on a continuous scale may thus be more effective — and more likely to influence decisions — than presenting them as categories (for example, ‘very low’ and ‘high’).

Third, the authors have found consistent evidence to suggest that GPs’ interpretation of illness duration runs counter to STARWAVe. Qualitative work investigating why GPs prescribe antibiotics for longer (versus shorter) illnesses could return valuable insights. GPs may be adopting a ‘wait-and-see’ approach to prescribing in an attempt to reduce prescriptions.^14^ Alternatively, they may be concerned that a prolonged infection has/will become bacterial, even if it began as viral. Either way, decision aid developers need to be aware that this risk factor is counterintuitive to GPs and may require explanation.

The present study highlights the importance of examining how practitioners appraise information and assess risk when designing decision support tools. When discrepancies are observed, such tools should be introduced carefully and explained to practitioners so that they do not result in loss of trust and limited use.

## Data

The data have been included in the submission and will be made publicly available on the Open Science Framework on publication.
